# Isolation of neutralizing antibodies against SARS-CoV-2 through an epitope-guided negative screening by phage display

**DOI:** 10.7555/JBR.39.20250259

**Published:** 2026-03-19

**Authors:** Ming Lu, Yin Chen, Xiaoyu Liu, Fang Gao, Liming Gou, Wei Ye, Jiaqi Wen, Xiling Guo, Wei Gao

**Affiliations:** 1 National Health Commission Key Laboratory of Antibody Technique, School of Basic Medical Sciences, Animal Core Facility of Nanjing Medical University, Nanjing Medical University, Nanjing, Jiangsu 211166, China; 2 Key Laboratory of Enteric Pathogenic Microbiology; Ministry of Health Institute of Pathogenic Microbiology; Jiangsu Province Center for Disease Control and Prevention, Nanjing, Jiangsu 211166, China; 3 The Second People's Hospital of Changzhou, the Third Affiliated Hospital of Nanjing Medical University, Changzhou, Jiangsu 213000, China

**Keywords:** epitope-guided, neutralizing antibody, SARS-CoV-2, phage display

## Abstract

Neutralizing antibodies are essential tools in antiviral therapy and epidemic preparedness, capable of directly inhibiting viral entry and limiting disease progression. However, traditional antibody discovery strategies—such as animal immunization or B cell isolation from infected individuals—are often hindered by biosafety concerns, lengthy development timelines, and limited adaptability during outbreaks. In the present study, we aimed to establish a robust and rapid *in vitro* platform for the efficient isolation of neutralizing antibodies targeting conserved viral epitopes. We developed an epitope-guided negative screening strategy that integrates phage display technology with rational antigen mutagenesis to exclude antibodies against variable regions while enriching for those that recognize functionally constrained epitopes. When applied to the receptor-binding domain of severe acute respiratory syndrome coronavirus 2, this method enabled the identification of six neutralizing antibodies (one IgG and five nanobodies) exhibiting broad-spectrum neutralizing activity across multiple viral variants. Notably, antibodies recognizing distinct epitopes demonstrated significant synergistic neutralization when used in combination (*P* < 0.05). This screening approach facilitates the rapid discovery of potent and mutation-resistant antibodies and holds promise for application to other emerging pathogens. Our findings underscore the potential of epitope-guided, *in vitro* platforms in expediting therapeutic antibody development under conditions of high biosafety requirements.

## Introduction

Therapeutic antibodies are widely used in the clinical treatment of cancer, autoimmune diseases, and infectious diseases^[1–4]^. Therapeutic antibodies that target pathogens can be categorized into neutralizing antibodies and non-neutralizing antibodies^[4–5]^. Neutralizing antibodies typically refer to those that recognize endogenous or infection-derived soluble antigens and exhibit a blocking effect on the phenotype induced by the antigen. A representative example is the virus-neutralizing antibody, which directly binds to critical epitopes on viral particles and thereby blocks cellular invasion or prevents viral membrane fusion of the virus^[6–7]^.

In the human body, the natural immune system is quickly activated by antigen stimulation and produces billions of antibodies. Based on this principle, various technologies have been employed to obtain therapeutic monoclonal antibodies^[8–11]^. These include high-throughput hybridoma screening, in which rodents are immunized^[12–13]^; display technologies such as phage or yeast display, which obtain the antigen binders *in vitro* by screening antibody libraries constructed from B cells isolated from naïve or immunized individuals^[14–16]^; and the recently developed single B-cell sorting strategy, which directly identifies antibody sequences by sequencing B cells isolated from infected individuals^[17–18]^.

Although hybridoma technology and single B cell sorting strategies may relatively easily yield antibodies, they involve lengthy procedures, high costs, and biosafety challenges, especially when responding to acute infectious diseases. More critically, these conventional approaches often prioritize antibodies against immunodominant but variable epitopes, leaving therapeutic candidates vulnerable to viral escape mutants in highly mutable pathogens like severe acute respiratory syndrome coronavirus 2 (SARS-CoV-2)^[19]^. In this context, display technology has unique advantages for enabling safe, economical, and efficient *in vitro* antibody screening. When targeting conserved functional sites essential for viral pathogenicity—such as those blocking ligand-receptor interactions or neutralizing pathogens—our strategy of epitope-guided phage display offers a distinct advantage: By incorporating antigen mutagenesis-driven negative screening, we actively deplete binders to mutable regions, thereby enriching for antibodies against evolutionarily constrained epitopes with a lower risk of viral escape^[20]^. When essential functional sites or domains of an antigen—such as those involved in blocking ligand-receptor interactions, neutralizing pathogens, modulating receptor activity, enhancing inflammatory responses, and recruiting proteins to form complexes—are identified^[18,21–22]^, the development of an epitope-guided phage display strategy for antibody screening may improve the selection specificity and efficiency. This makes the method suitable for various targets, including acute infectious diseases, cancer, and autoimmune disorders, significantly accelerating the development of therapeutic antibodies^[23]^.

SARS-CoV-2, the causative agent of Coronavirus Disease 2019 (COVID-19), enters host cells *via* angiotensin-converting enzyme 2 (ACE2) and activates innate immune responses, including Toll-like receptor (TLR)-mediated pathways^[24–25]^. This often leads to excessive cytokine release and immunopathogenesis, such as acute lung injury. Despite the use of antivirals, immunosuppressants, and vaccines, emerging variants continue to challenge current treatments. Therefore, monoclonal antibodies targeting conserved viral epitopes remain a promising and adaptable therapeutic strategy^[26]^.

In the present study, we aimed to establish an epitope-guided phage display strategy for isolating neutralizing antibodies against SARS-CoV-2. To this end, we engineered a mutant receptor-binding domain (RBD) antigen with a disrupted receptor-binding motif (RBM) to serve as a negative screening agent for the precise enrichment of target antibodies.

## Materials and methods

### Plasmids and reagents

The coding sequences of ACE2 and SARS-CoV-2 spike protein were cloned into the pLVX vector to construct stable cell lines. The coding sequences of truncated ACE2 (Q18-S740), SARS-CoV-1-RBD (P317-V510), SARS-CoV-2-RBD (P330-V524), and the SARS-CoV-2-RBD mutant were cloned into the PFUSE vector to generate hFc-fusion proteins. The SARS-CoV-2-RBD mutant was designed by substituting key residues in SARS-CoV-2-RBD with alanine (Ala) or phenylalanine (Phe) to disrupt its interaction with ACE2. The coding sequences of domain-specific antibodies were cloned into the PFUSE vector to generate hFc-fusion proteins. The coding sequences of the 4A3 heavy chain variable region and light chain variable region were amplified with the addition of an IL-2 signal peptide and cloned into the expression vectors PFUSE-CHIg-HG1 and PFUSE-CLIg-hk (InvivoGen, San Diego, CA, USA), respectively. All plasmids were verified by sequencing. The SARS-CoV-2-RBD-His protein and GPC5-His protein were purchased from GenScript (Nanjing, Jiangsu, China) and R&D Systems (Minneapolis, MN, USA), respectively.

### Cell culture

HEK293T and Huh-7 cells were purchased from the American Type Culture Collection (ATCC, Rockville, MD, USA). CHO-K1 cells were kindly provided by the School of Basic Medical Sciences, Nanjing Medical University (Nanjing, Jiangsu, China). Vero E6 cells were purchased from the American Type Culture Collection (ATCC). ACE2-CHO and SARS-CoV-2-spike-CHO stable cell lines were generated in our laboratory as described above. All cells were cultured in Dulbecco's Modified Eagle's Medium (DMEM; Servicebio, Wuhan, Hubei, China) supplemented with 10% fetal bovine serum (Gibco, Carlsbad, CA, USA) and 1% penicillin-streptomycin solution (10000 U/mL penicillin, 10 mg/mL streptomycin; Servicebio) at 37 ℃ in a humidified atmosphere containing 5% CO_2_. All cell lines were passaged for fewer than 10 passages during the experiments and were confirmed to be free of mycoplasma contamination by PCR testing prior to use.

### Structural modeling

The structure files of SARS-CoV-1/ACE2 complex (PDB ID: 2AJF), SARS-CoV-2-RBD/ACE2 complex (PDB ID: 6M0J), ectodomain of SARS-CoV-2 spike trimer in the closed (PDB ID: 6VXX) and open (PDB ID: 6VYB) states were downloaded from the RCSB Protein Data Bank. The structural model of SARS-CoV-2-RBD mutant was predicted by the Protein Fold Recognition Server Phyre2. The remodeled ectodomain trimer of SARS-CoV-2 spike in both open and closed states was established by replacing the partially determined RBD of structures (PDB IDs: 6VYB for the open state and 6VXX for the closed state) with the fully determined SARS-CoV-2-RBD (PDB ID: 6M0J) using PyMol, Discovery Studio, and SWISS-MODEL.

### Phage display

The TG1 clone was picked and cultured overnight in 2YT medium at 37 ℃. SARS-CoV-2-RBD-His or SARS-CoV-2-RBD-hFc proteins in PBS buffer were coated on an enzyme-linked immunosorbent assay (ELISA) plate at 4 ℃ overnight. The Tomlinson Ⅰ phage display library (library size: 1.47 × 10^8^ independent scFv clones) and the nanobody library (library size: 1.19 × 10^8^ CFU, estimated from standard construction protocols) were pre-blocked with PBST containing 5% skim milk at room temperature for 1 h. The blocked phages were pre-cleared with negative antigens and then added to the coated wells and incubated at room temperature for one hour. After washing 20 times with PBST, the SARS-CoV-2-RBD-binding phages were eluted with 100 mmol/L triethylamine (Sigma-Aldrich, Xuhui, Shanghai). All eluted phages were collected and used to infect TG1 cells. After incubation with helper phages, the eluted phages were rescued with a titer of approximately 10^11^–10^12^ plaque-forming units per milliliter (PFU/mL) for the next round of panning.

### ELISA

For direct ELISA, indicated antigens (5 μg/mL) were coated on ELISA plates at 4 ℃ overnight. After blocking, biotin-labeled ACE2, blocked phages, or indicated antibodies were added to the wells and incubated at 37 ℃ for 0.5 h. Streptavidin-HRP (Thermo Fisher Scientific, Waltham, MA, USA), rabbit anti-M13 HRP antibody (for phage) (GE Healthcare, Milwaukee, WI, USA), or goat anti-human Fcγ HRP antibody (Jackson ImmunoResearch, West Grove, PA, USA) was added. TMB and H_2_SO_4_ were added to detect the optical density (OD) at 450 nm. For capture ELISA, anti-His antibody (5 μg/mL) was coated on the ELISA plate at 4 ℃ overnight. After blocking, soluble antibodies extracted from the periplasm of TG1 cells were added to the wells and incubated at 37 ℃ for 0.5 h. After washing, SARS-CoV-1-RBD-hFc, SARS-CoV-2-RBD-hFc, or SARS-CoV-2-RBD mutant-hFc proteins (5 μg/mL) were added and incubated at 37 ℃ for 0.5 h. After washing, goat anti-human Fcγ HRP antibody (Jackson ImmunoResearch) was added and incubated at 37 ℃ for 0.5 h. TMB and H_2_SO_4_ were added to detect the OD at 450 nm.

### Cell binding and antibody blocking assay

For the cell binding assay, a single-cell suspension of SARS-CoV-2-spike-CHO cells was incubated with 5 µg/mL of indicated antibodies for 1 h on ice, and then incubated with goat anti-human PE antibody diluted 1∶200 (Thermo) for 1 h on ice. The cells were analyzed using a Fluorescence-Activated Cell Sorter (FACS) Calibur instrument (BD Biosciences, San Jose, CA, USA). For the antibody blocking assay, antibodies were pre-incubated with 2.5 µg/mL of SARS-CoV-1-RBD-hFc or SARS-CoV-2-RBD-hFc at different concentrations for 1 h on ice, and then the mixture was incubated with ACE2-CHO cells for 1 h on ice. After washing, goat anti-human PE antibody (Thermo) was added to cells at a 1∶200 dilution and incubated for 1 h on ice. The cells were analyzed by FACS Calibur (BD Biosciences).

### Surface plasmon resonance (SPR) analysis

SPR analysis was performed by GenScript (GenScript, Nanjing, China) to measure the affinity of antibodies. Antibodies were immobilized on the Series S Sensor Chip Protein A chip (GE Healthcare), and then SARS-CoV-2-RBD-His protein with a gradient concentration of 1.25 to 40 nmol/L was injected into the chip. The analysis was performed at a constant temperature of 25 ℃. The buffer was HBS-EP^+^: 10 mmol/L HEPES, 150 mmol/L NaCl, 3 mmol/L EDTA, 0.05% P20, pH 7.4 (Cat. #30393; GE Healthcare); the flow rate was 10 μL/min. The assay was performed using a Biacore T200, GR18010468 (GE Healthcare). The binding kinetics and affinity (equilibrium dissociation constant, KD) were calculated using Biacore T200 Evaluation software version 3.1.

### Pseudovirus neutralization assay

The SARS-CoV-2 strain used in the present study belongs to Pango lineage B.1.2, as determined by genomic sequence analysis using Nextclade (version 3.16.0). To generate the SARS-CoV-2 pseudovirus, we replaced the coding sequence of vesicular stomatitis virus glycoprotein (VSV-G) protein with the sequence of SARS-CoV-2 spike in the lentiviral packaging system, and then co-transfected HEK293T cells with the pLVX-EGFP-Luciferase reporter gene. The pseudovirus supernatant was collected 48 h later and titered to 10^5^ PFU/mL. Neutralization assays were performed by incubating the pseudovirus with serially diluted antibodies at 37 ℃ for 1 h. The pseudovirus-antibody mixture was then added to seeded ACE2-CHO cells (approximately 5 × 10^3^ PFU and 10^4^ cells/well) in 96-well plates. The half-maximal inhibitory concentration (IC_50_) of each antibody was determined by measuring luciferase activity 48 h later. Antibody neutralization titers were determined using a two-fold serial dilution method. The IC_50_ and half-maximal effective concentration (EC_50_) values were calculated by fitting the dose-response data with a four-parameter logistic (4-PL) nonlinear regression model. All IC_50_ and EC_50_ values were log_10_-transformed prior to statistical analysis to approximate a normal distribution and meet the assumptions of parametric tests.

### Live SARS-CoV-2 neutralization assay

All experiments with live SARS-CoV-2 were performed under the approved standard operating procedures of the Biosafety Level 3 laboratory. The live SARS-CoV-2 viruses were isolated from the throat swab of the SARS-CoV-2-infected patient in Jiangsu Province and identified by sequencing. The virus was amplified in Vero E6 cells and stored as working stocks at 10^5^ PFU/mL. For the neutralization assay, Vero E6 cells were seeded into a 96-well plate at 10^4^/well and cultured overnight. SARS-CoV-2 viruses at 100 tissue culture infective dose 50% (TCID_50_) were pre-incubated with serially diluted antibodies at 37 ℃ for 1 h. The virus-antibody mixture was then added to seeded Vero E6 cells. Cytopathic effects (CPE) were photographed four days later.

### Statistical analysis

Data were presented as the mean ± standard deviation of a representative experiment performed with at least three technical replicates. Similar results were obtained in at least three independent experiments (*n* = 3). Comparisons between two groups were performed using an unpaired, two-tailed Student's *t*-test. For comparisons among more than two groups, one-way analysis of variance (ANOVA) was employed, followed by Tukey's post hoc test for multiple comparisons. All statistical analyses were performed using GraphPad Prism version 8.0 (GraphPad Software, San Diego, CA, USA), and *P*-values < 0.05 were considered statistically significant.

## Results

### Purification of SARS-CoV-2-RBD mutant protein with a disrupted ACE2 interface to abolish ACE2 binding

**Figure 1:**
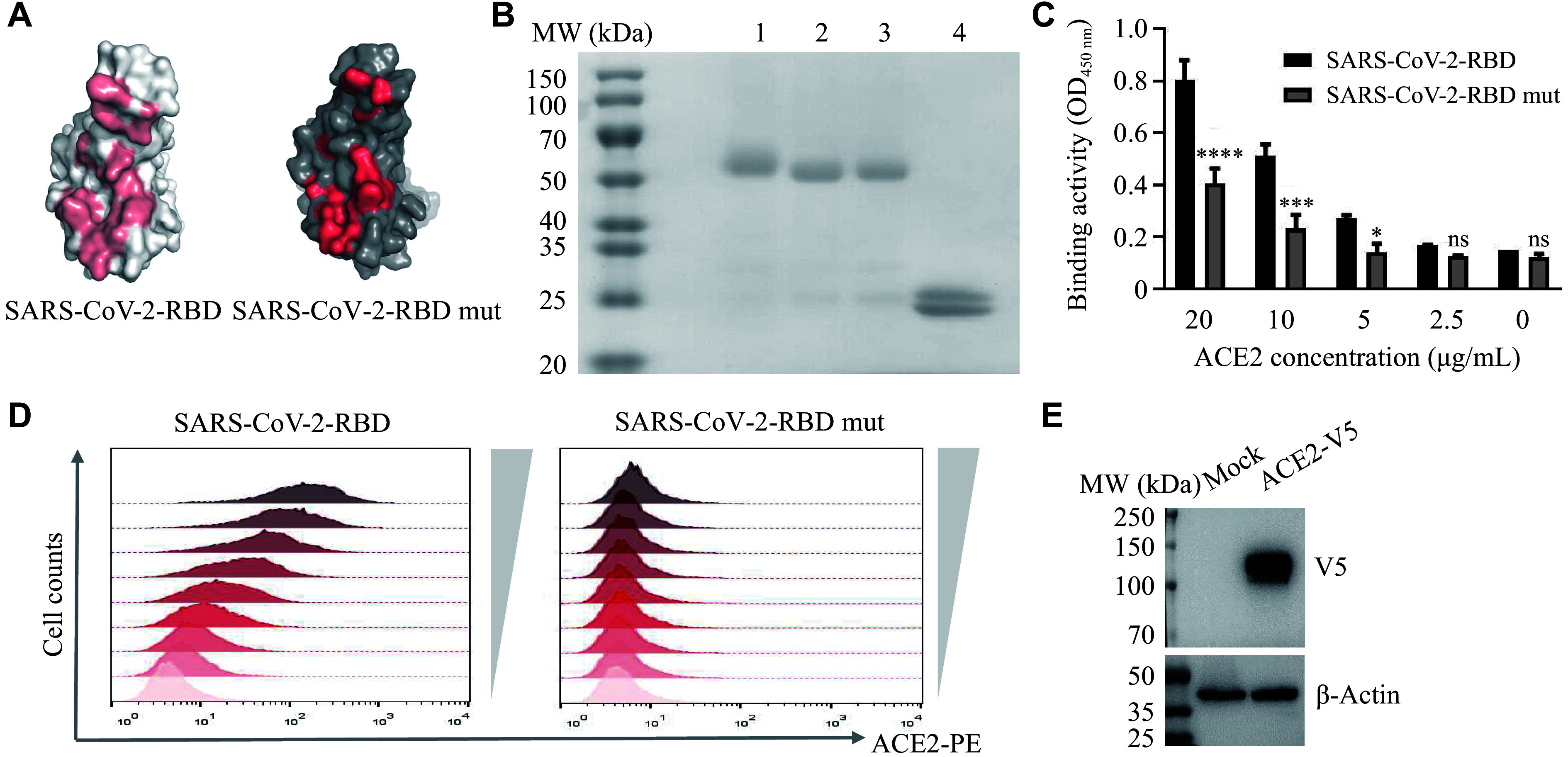
Design, preparation, and verification of SARS-CoV-2-RBD antigens. A: Molecular surface representation of the ACE2 interfaces of wild-type (WT) SARS-CoV-2-RBD and the RBD mutant. B: SDS-PAGE analysis of the RBD proteins under reducing conditions. Lanes: 1, SARS-CoV-1-RBD-hFc; 2, SARS-CoV-2-RBD mut-hFc; 3, SARS-CoV-2-RBD-hFc; 4, SARS-CoV-2-RBD-His. C and D: Binding of serially diluted WT and mutant RBD proteins to ACE2 at the protein level by ELISA (C) and at the cell level by flow cytometry (D). E: Western blot analysis of V5-tagged ACE2 expression in stably transfected CHO-K1 cells. Data are presented as mean ± standard deviation (*n* = 3). Statistical significance was determined by unpaired Student's *t*-test. ^*^P < 0.05, ^***^P < 0.001, and ^****^P < 0.0001 compared with the SARS-CoV-2 RBD group. Abbreviations: ACE2, angiotensin-converting enzyme 2; OD, optical density; ELISA, enzyme-linked immunosorbent assay; ns, not significant; RBD, receptor-binding domain; SARS-CoV-2, severe acute respiratory syndrome coronavirus 2; SDS-PAGE, sodium dodecyl sulfate-polyacrylamide gel electrophoresis.

To obtain antibodies targeting SARS-CoV-2-RBD, we selected sixteen residues essential for the hydrophobic or electrostatic interactions within the ACE2 interface of SARS-CoV-2 and introduced mutations into these residues. The predicted structural model of SARS-CoV-2-RBD mutant showed that the overall conformation of the RBD did not change after mutation (***Supplementary Fig. 1***), but the surface properties of the ACE2 interface were altered (***Fig. 1A***). We then designed recombinant proteins for SARS-CoV-2-RBD, SARS-CoV-2-RBD mutant, and SARS-CoV-1-RBD, each fused with a human Fc tag, and subsequently performed protein purification (***Fig. 1B***). The recombinant SARS-CoV-2-RBD mutant protein exhibited impaired binding activity to ACE2 protein and ACE2-CHO cells compared with the wild-type (***Fig. 1C*** and ***1D***). The expression of ACE2 in the CHO-K1 cell line used for binding assays was confirmed by Western blotting (***Fig. 1E***). These results demonstrated that these mutations successfully abolished ACE2 recognition by disrupting the ACE2 interface of SARS-CoV-2-RBD. The purified SARS-CoV-2-RBD mutant was therefore suitable for use as the negative antigen in antibody screening.

### Isolation of SARS-CoV-2-specific antibody by *in vitro* epitope-guided negative screening

**Figure 2:**
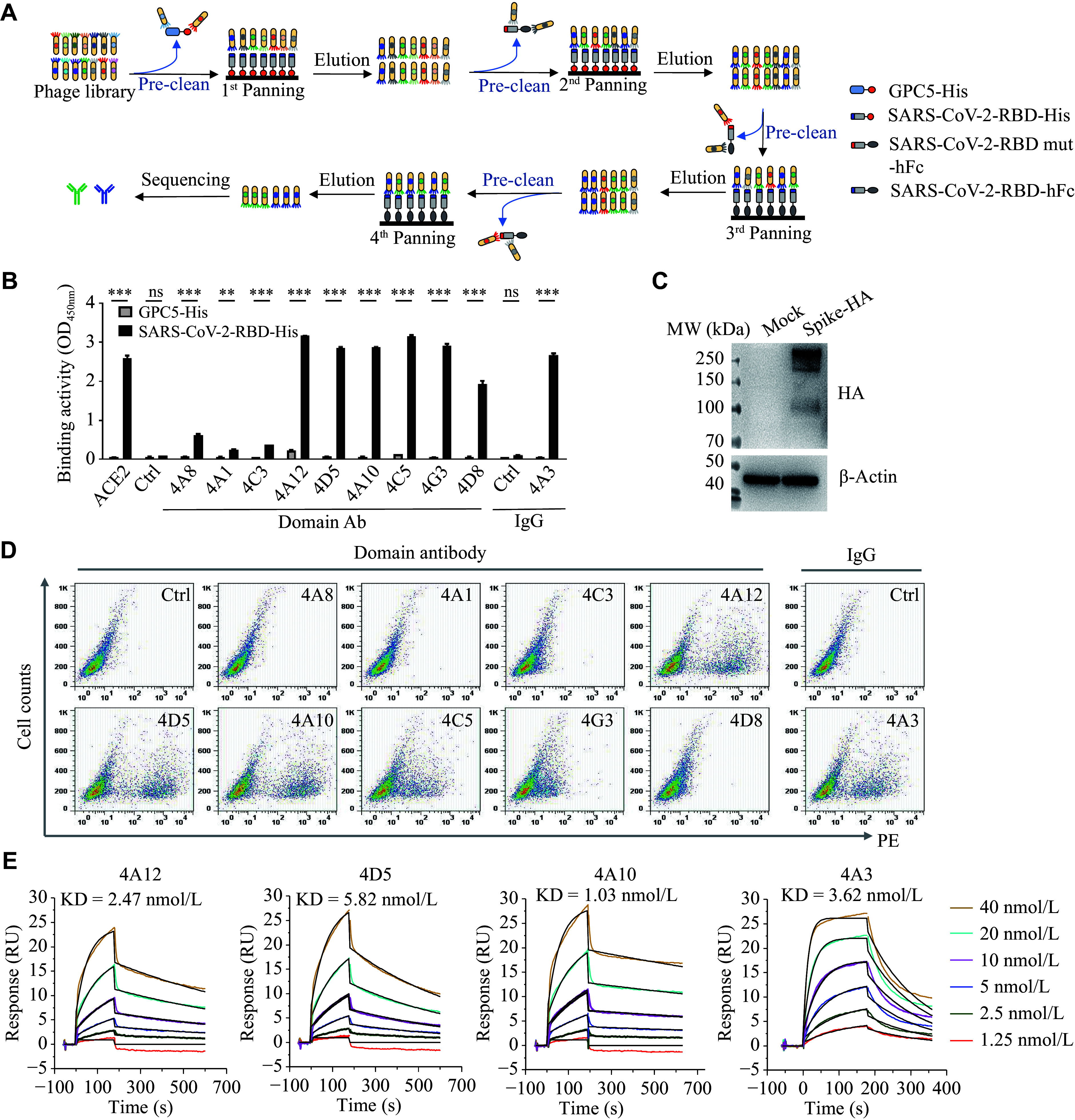
Antibody screening by phage display. A: Schematic of the SARS-CoV-2-RBD-binding antibody screening process using phage display, featuring a pre-adsorption step against GPC5-his and SARS-CoV-2-RBD mutant-Fc to deplete non-specific and off-target binders. B: Polyclonal phage ELISA showing the SARS-CoV-2-RBD-his binding activity of phages from four rounds of rescue. GPC5-his was used as a negative antigen control. C: Western blot analysis of SARS-CoV-2 spike protein expression in stably transfected CHO-K1 cells. D: Flow cytometry analysis of domain antibodies and IgG antibody binding to SARS-CoV-2 spike protein-expressing cells. All dot plots share identical axis ranges (X: PE fluorescence 10^0^–10^4^; Y: Cell Counts 10^0^–10^3^). E: Surface plasmon resonance (SPR) sensograms measuring the binding affinity of SARS-CoV-2-RBD-his protein to captured antibodies. Data are presented as the mean ± standard deviation (*n* = 3). Statistical significance was determined by one-way analysis of variance (ANOVA) with Tukey's multiple comparisons test. ^**^P < 0.01 and ^***^P < 0.001. Abbreviations: ELISA, enzyme-linked immunosorbent assay; Fc, fragment crystallizable region; His, polyhistidine tag; ns, not significant; PE, phycoerythrin; RBD, receptor-binding domain; SARS-CoV-2, severe acute respiratory syndrome coronavirus 2; SPR, surface plasmon resonance.

To obtain SARS-CoV-2-specific neutralizing antibodies, we performed epitope-guided negative screening by phage display. We used SARS-CoV-2-RBD-His and SARS-CoV-2-RBD-hFc as positive antigens and GPC5-His and SARS-CoV-2-RBD mutant-hFc as negative antigens to perform selection from a naive human scFv antibody phage library and a nanobody phage library, respectively (***Fig. 2A***). After four rounds of screening, the antigen-binding activity of the eluted phages dramatically increased (***Supplementary Fig. 2A***). Notably, the eluted phages exhibited stronger binding signals for SARS-CoV-2-RBD than for SARS-CoV-2-RBD mutant, especially those from the nanobody library (***Supplementary Fig. 2B***), indicating an expected precleaning effect during selection. We then randomly picked 200 single clones from the fourth-round eluted phages and performed monoclonal phage ELISA. The positive binders were significantly enriched in both libraries (***Supplementary Fig. 2C***). All the positive binders were sequenced, and finally, we obtained nine enriched clones from the nanobody library and one enriched clone from the scFv antibody library (***Supplementary Table 1***). Among them, nine domain antibodies bound specifically to SARS-CoV-2-RBD, whereas one scFv antibody (4A3) showed weak binding activity to SARS-CoV-1-RBD and SARS-CoV-2-RBD mutant in the phage ELISA (***Supplementary Fig. 2D***).

The nine binders isolated from the nanobody library contained only the antibody heavy chain variable region. We then fused them with a human Fc tag and performed protein purification. The 4A3 scFv binder was converted into a human IgG1 format and purified as well (***Supplementary Fig. 3***). Among all the purified antibodies, 4A12, 4D5, 4A10, 4C5, and 4A3 were selected for further evaluation because of their strong binding activities to both SARS-CoV-2-RBD (***Fig. 2B***) and SARS-CoV-2 spike-overexpressing cells (***Fig. 2C*** and ***2D***), as well as their promising high expression yield and purity.

We then performed SPR analysis to further evaluate the affinities of these candidate antibodies. The affinities of our antibodies ranged from 1.03 nmol/L to 5.82 nmol/L (***Fig. 2E***), with detailed kinetic parameters shown in ***Supplementary Table 2***. Altogether, these results indicated that we obtained multiple antibodies with potential neutralizing activity against SARS-CoV-2.

### The candidate antibodies blocked the binding of SARS-CoV-2-RBD to ACE2-positive cells

**Figure 3:**
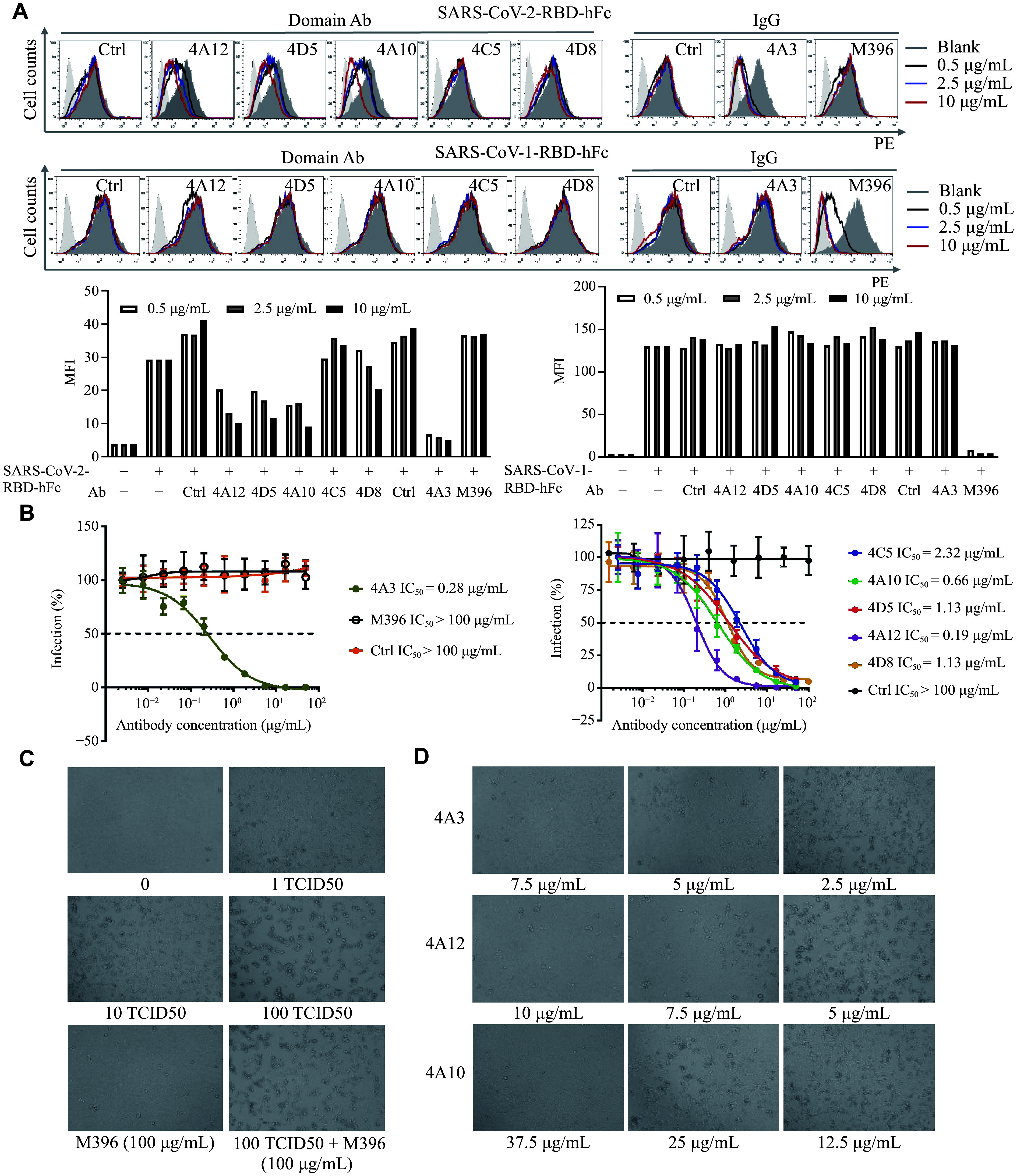
The binding properties of domain antibodies and IgG antibodies. A: Flow cytometry analysis of the blocking effect of domain antibodies and IgG antibodies on the binding of SARS-CoV-2-RBD-hFc (upper row) and SARS-CoV-1-RBD-hFc (lower row) to ACE2-CHO cells. Cells were treated with the indicated concentrations of antibodies, using the M396 antibody as a negative control. The histogram shows the analysis of the mean fluorescence intensity (MFI) from a single representative experiment. The MFI values were derived from the entire cell population analyzed in the experiment, demonstrating a clear and reproducible blocking trend for the candidate antibodies. B: Neutralization effects of candidate antibodies against SARS-CoV-2 pseudovirus. Data are presented as mean ± standard deviation from three independent experiments (*n* = 3). The 32A9 IgG (targeting glypican-3) and M396 antibody were used as controls for the 4A3 IgG. The nanobody 31A2 (against galectin-3) was used as the control nanobody. Neutralization potency is represented by the half-maximal inhibitory concentration (IC_50_). C: Quality control of live SARS-CoV-2 infectivity. Representative images of the cytopathic effect (CPE) in Vero E6 cells exposed to 1 TCID_50_, 10 TCID_50_, and 100 TCID_50_ of SARS-CoV-2 for four days. The M396 antibody was used as a control. D: Neutralization of live SARS-CoV-2 by candidate antibodies. Vero E6 cells were infected with SARS-CoV-2 (100 TCID_50_) that had been pre-incubated with the indicated antibodies. Representative images of the CPE were taken four days post-infection. Antibody concentrations (7.5, 10, and 37.5 µg/mL for 4A3, 4A12, and 4A10, respectively) indicating complete protection are labeled. Statistical significance for the dose-response curves in B was determined from three independent experiments (*n* = 3) by two-way ANOVA with Tukey's multiple comparisons test. Abbreviations: ACE2, angiotensin-converting enzyme 2; hFc, human fragment crystallizable region; IC_50_, half-maximal inhibitory concentration; IgG, immunoglobulin G; RBD, receptor-binding domain; SARS-CoV-2, severe acute respiratory syndrome coronavirus 2; TCID_50_, 50% tissue culture infectious dose.

To examine the potential neutralizing capabilities of our candidate antibodies, we investigated whether they would disturb the binding between SARS-CoV-2-RBD and ACE2-positive cells. Three domain antibodies (4A12, 4D5, and 4A10) and the 4A3 IgG exhibited obvious inhibition in a dose-dependent manner, whereas the SARS-CoV-1-neutralizing antibody M396 showed no effect. Considering the high similarity of the RBDs, we also evaluated the blocking effects of our antibodies on the binding between SARS-CoV-1-RBD and ACE2-CHO cells. None of our candidate antibodies showed inhibitory effects (***Fig. 3A***). Notably, we suspected that clone 4A3, which showed weak cross-reactivity with SARS-CoV-1-RBD in the phage ELISA (***Supplementary Fig. 2D***), might exhibit a certain blocking effect on the cell binding of SARS-CoV-1-RBD; however, it specifically blocked only the cell binding of SARS-CoV-2-RBD.

To evaluate the potential antiviral activities of our antibodies, we prepared a SARS-CoV-2 pseudovirus by replacing the coding sequence of VSV-G with SARS-CoV-2 spike glycoprotein in a lentivirus packaging system. The domain antibodies 4A12, 4A10, 4D5, and 4A3 IgG exhibited potent neutralizing activity, with IC_50_ values ranging from 0.19 µg/mL to 1.13 µg/mL (***Fig. 3B***). These results were consistent with the blocking pattern observed in the SARS-CoV-2-RBD and ACE2-CHO cell-binding assays (***Fig. 2D***). Although 4C5 did not show a blocking effect in our cell-binding assay, it still exhibited a mild neutralizing capability in this assay. Based on our pseudovirus experiment, we selected 4A3 IgG, 4A12, and 4A10 to evaluate their neutralizing activities against live SARS-CoV-2. The CPE assay revealed that 4A3, 4A12, and 4A10 exhibited complete protection at 7.5 µg/mL, 10 µg/mL, and 37.5 µg/mL, respectively, after four days of SARS-CoV-2 exposure (100 TCID_50_; ***Fig. 3C*** and ***3D***). These protective effects remained highly stable when the exposure time was extended to 10 to 15 days (***Supplementary Table 3***). These findings underscore the efficiency and precision of our antibody screening method in identifying neutralizing antibodies targeting specific epitopes, which are particularly relevant for acute infectious diseases.

### Epitope mapping of SARS-CoV-2-RBD candidate antibodies

**Figure 4:**
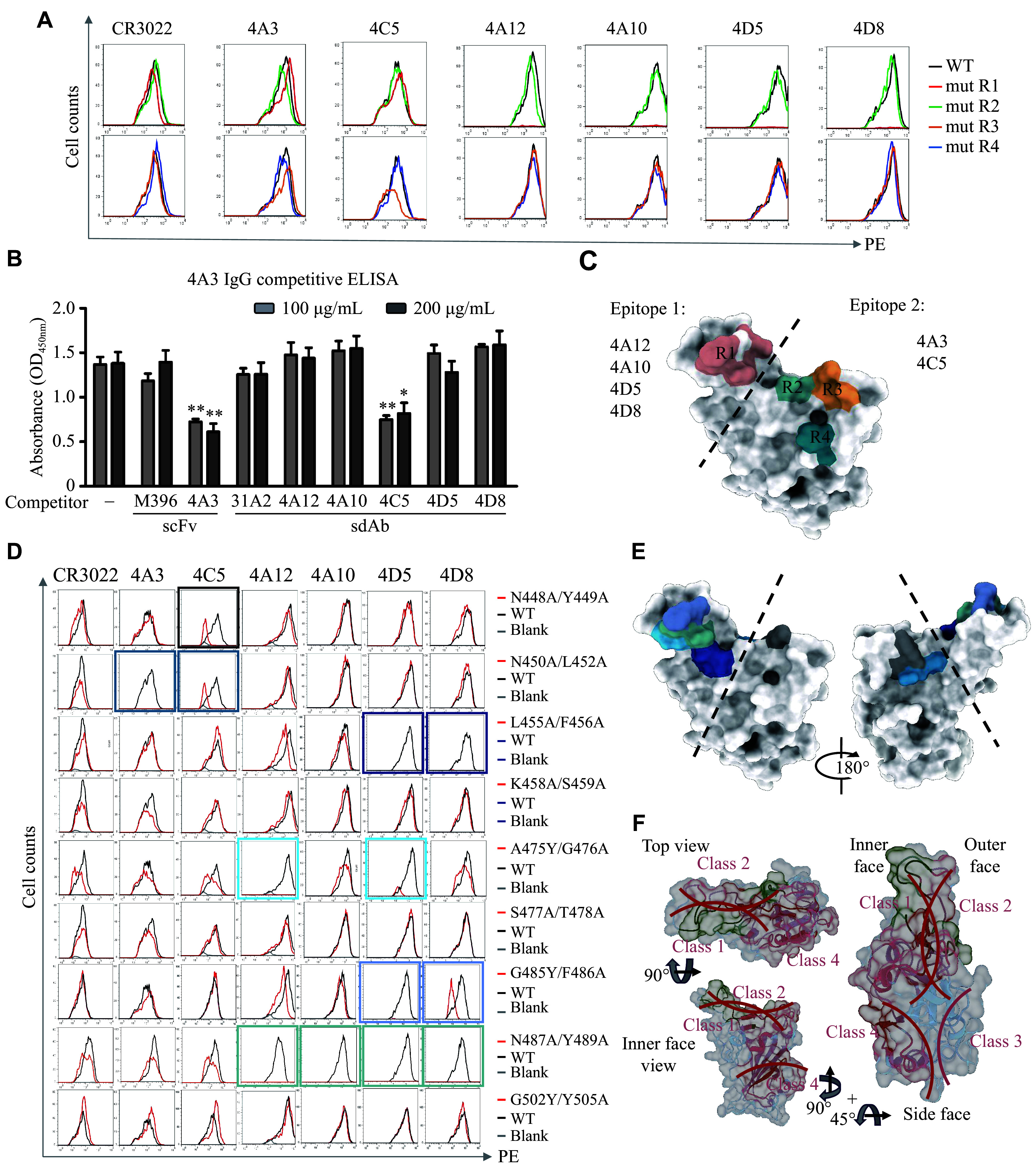
Binding properties of the domain antibodies and the IgG antibody. A and B: To determine the critical residues for antibody recognition, the binding of candidate antibodies to a panel of SARS-CoV-2-RBD mutant proteins was assessed at the cellular level by flow cytometry (A) and at the protein level by ELISA (B). C: The modeled structures of SARS-CoV-2-RBD mutant region, domain antibodies, and IgG antibody recognition regions were categorized into two groups. D: Validation of predicted antibody recognition regions by flow cytometry using RBD proteins with point mutations in key epitope regions. E: Model depicting the spatial distribution of the predicted epitopes for antibody recognition on the SARS-CoV-2-RBD. F: Graphic depiction of the number of contacts illustrated as a footprint on the RBD and as a putty heat map of the RBD cartoon backbone. Top, inner face, and side views of RBD are shown. Data are presented as the mean ± standard deviation (*n* = 3). Statistical significance was determined by one-way analysis of variance (ANOVA) with Tukey's multiple comparisons test. ^*^P < 0.05 and ^**^P < 0.01 indicate significant differences compared with the non-competing control group. Abbreviations: ELISA, enzyme-linked immunosorbent assay; IgG, immunoglobulin G; mut, mutant; RBD, receptor-binding domain; SARS-CoV-2, severe acute respiratory syndrome coronavirus 2; scFv, single-chain variable fragment; sdAb, single-domain antibody; WT, wild-type.

To investigate the specific epitopes recognized by the antibodies, we divided the interaction surface of the SARS-CoV-2-RBD into four distinct regions. Point mutations were introduced into these four regions, and cell lines expressing the mutated RBD proteins were generated to analyze the distribution of antibody recognition epitopes. FACS analysis showed that all antibodies were able to recognize the mutated RBD proteins, but the binding affinity of antibodies 4A3 and 4C5 decreased significantly for the mutated R3 protein (***Fig. 4A***). Subsequently, antibody competition experiments revealed a competitive relationship between antibodies 4A3 and 4C5 (***Fig. 4B***), while no competition was observed between these two and the other antibodies. We categorized the recognized epitopes into two regions (***Fig. 4C***). To further refine the epitope mapping, we introduced single-point mutations at multiple positions on the SARS-CoV-2-RBD protein (***Supplementary Tables 4*** and ***5***) and expressed these mutated proteins on the cell surface. Additional FACS experiments helped pinpoint the regions of the RBD protein recognized by different antibodies (***Fig. 4D*** and ***4E***). To visualize the epitope distribution of our antibodies, we generated a surface heat map of the SARS-CoV-2-RBD (***Fig. 4F***). Red regions indicate residues targeted by more than 100 known antibodies^[27]^, while green indicates the epitopes of our antibodies. These epitopes are primarily located within the Class 1 and Class 2 regions, aligning with the major immunodominant sites targeted by most neutralizing antibodies. Based on these findings, we identified multiple antibodies, each targeting different epitopes on the RBD. Given these distinct recognition regions, these antibodies may be well-suited for multi-antibody combination therapies.

### The combined treatment of candidate antibodies exhibited synergistic neutralization effects against SARS-CoV-2 and its variants

**Figure 5:**
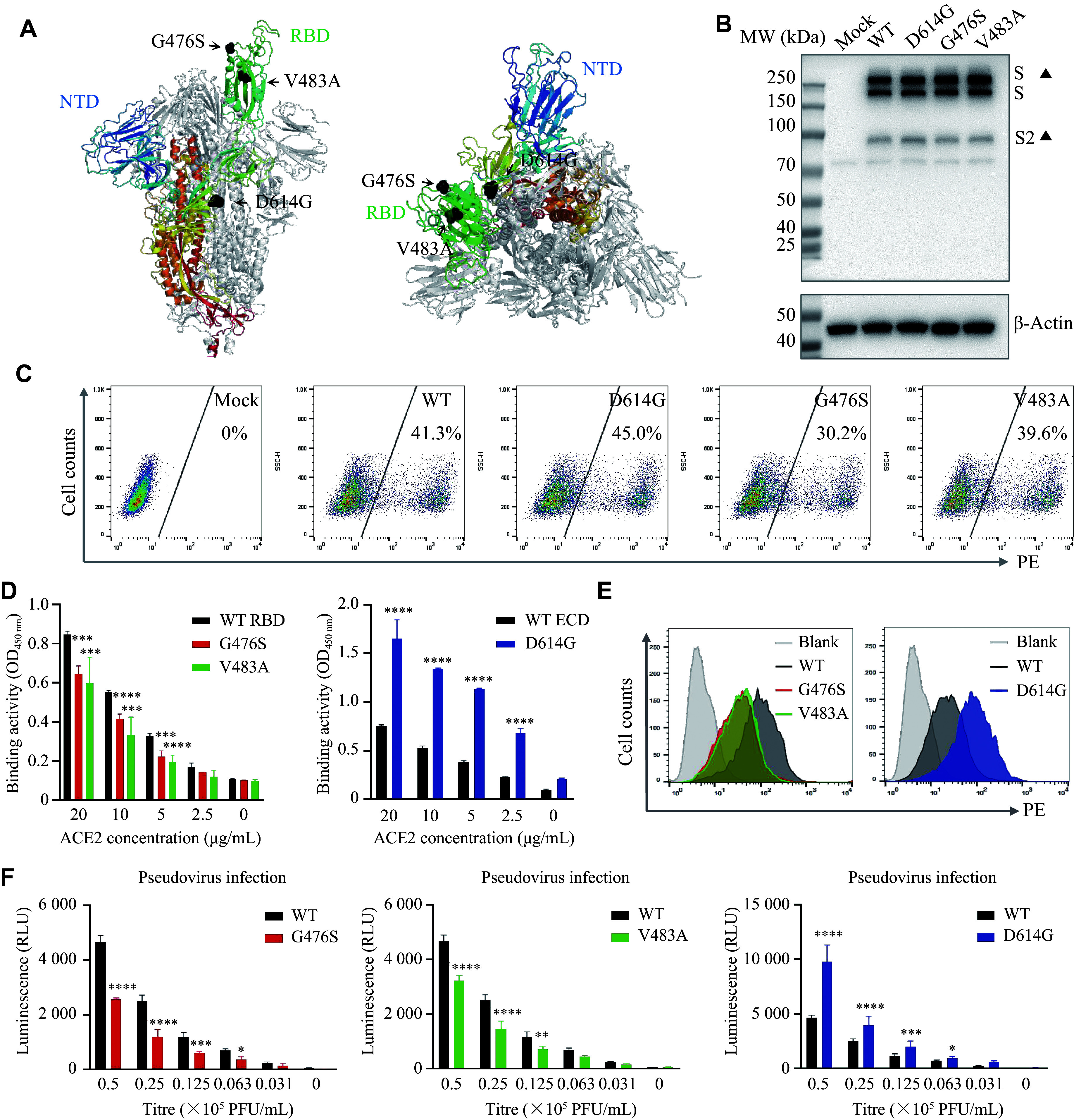
Shedding and characterization of SARS-CoV-2 variants. A: Structural models of the SARS-CoV-2 spike trimer (front and top views), highlighting the receptor-binding domain (RBD, green), the N-terminal domain (NTD, blue), and key mutation sites (black). B: SDS-PAGE analysis under reducing conditions of purified wild-type (WT) and mutant SARS-CoV-2 spike proteins. C: Flow cytometry analysis of the binding of WT and mutant spike proteins to ACE2-expressing CHO cells to assess the impact of mutations on cell surface ACE2 binding. D: ELISA assessing the binding of SARS-CoV-2 RBD variants (left) and SARS-CoV-2 ECD variants (right) to human ACE2. SARS-CoV-2-RBD-WT and SARS-CoV-2-ECD-WT were used as negative controls. E: Flow cytometry analysis of the binding of SARS-CoV-2 RBD variants (left) and SARS-CoV-2 ECD variants (right) to human ACE2 at the cellular level. Huh-7 cells (ACE2-negative) were used as a negative control. F: ELISA measuring the infectivity of pseudoviruses bearing the WT RBD versus a mutant RBD on ACE2-CHO cells. Data are presented as the mean ± standard deviation (*n* = 3). Statistical significance was determined by two-way ANOVA with Tukey's multiple comparisons test. ^*^P < 0.05, ^**^P < 0.01, ^***^P < 0.001, and ^****^P < 0.0001 indicate significant differences compared with the corresponding WT group. Abbreviations: ACE2, angiotensin-converting enzyme 2; ECD, extracellular domain; ELISA, enzyme-linked immunosorbent assay; SARS-CoV-2, severe acute respiratory syndrome coronavirus 2; SDS-PAGE, sodium dodecyl sulfate-polyacrylamide gel electrophoresis.

The high variability of pathogens causing acute infectious diseases allows pathogens, including SARS-CoV-2, to evade immune responses and diagnostic or therapeutic interventions through mutations, notably in the RBD region. We investigated SARS-CoV-2 spike protein variants, including D614G, G476S, and V483A (***Fig. 5A***). To assess antibody recognition, we expressed and purified these spike protein variants (***Fig. 5B***). Our results showed that the D614G variant had significantly higher ACE2 binding affinity (***Fig. 5C***–***5E***), and this increased binding correlated with enhanced pseudovirus infectivity (***Fig. 5F***).

**Figure 6:**
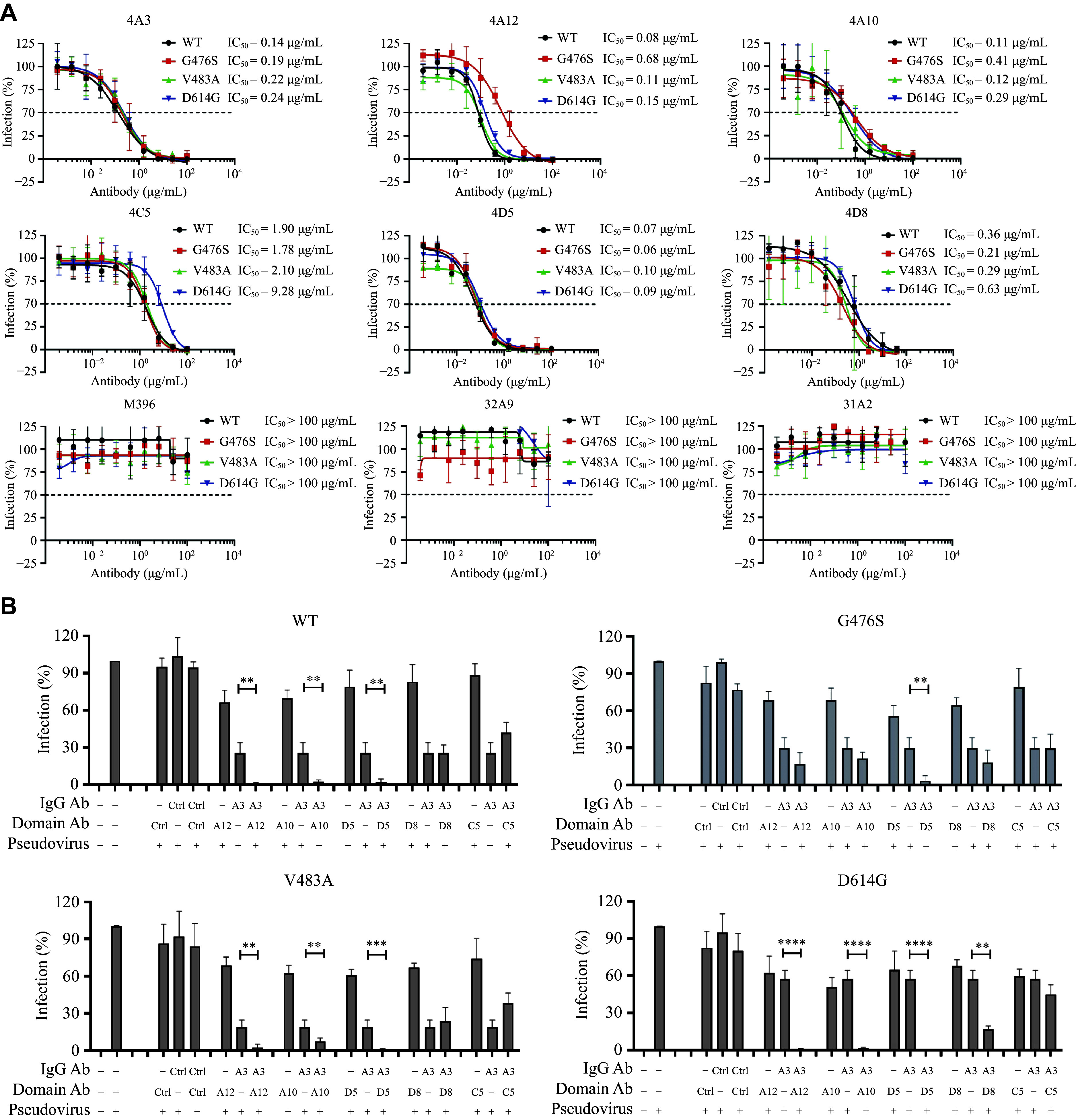
Antibodies reveal neutralizing effect on SARS-CoV-2 variants. A: Neutralization assay to detect the neutralizing activity of domain antibodies and IgG antibodies against SARS-CoV-2 spike and SARS-CoV-2 spike mutants. B: Assessment of competition and synergistic neutralization effects between domain antibodies (0.5 nmol/L) and 4A3 IgG antibody (0.5 nmol/L) on SARS-CoV-2 spike pseudovirus and SARS-CoV-2 spike mutant pseudovirus. Data are presented as the mean ± standard deviation (*n* = 3). Statistical significance was determined by two-way ANOVA with Tukey's multiple comparisons test. ^**^P < 0.01, ^***^P < 0.001, and ^****^P < 0.0001. Abbreviations: Ctrl, control; IgG, immunoglobulin G; SARS-CoV-2, severe acute respiratory syndrome coronavirus 2; WT, wild-type.

We evaluated the neutralizing activity of selected antibodies against these variants using pseudovirus assays. All antibodies retained neutralizing activity against the variants (***Fig. 6A***), indicating that single-point mutations did not affect antibody recognition of the spike protein. Since the screened antibodies recognized non-overlapping epitopes (***Fig. 4C*** and ***4E***), we explored their potential synergistic effects. Except for epitope-conflicting antibodies 4A3 and 4C5, the combined use of other antibodies exhibited synergistic effects in neutralizing SARS-CoV-2 (***Fig. 6B***).

In summary, we established a precise *in vitro* antibody screening strategy using SARS-CoV-2, efficiently identifying antibodies against specific epitopes. When combined, these antibodies showed enhanced neutralizing effects.

## Discussion

**Figure 7:**
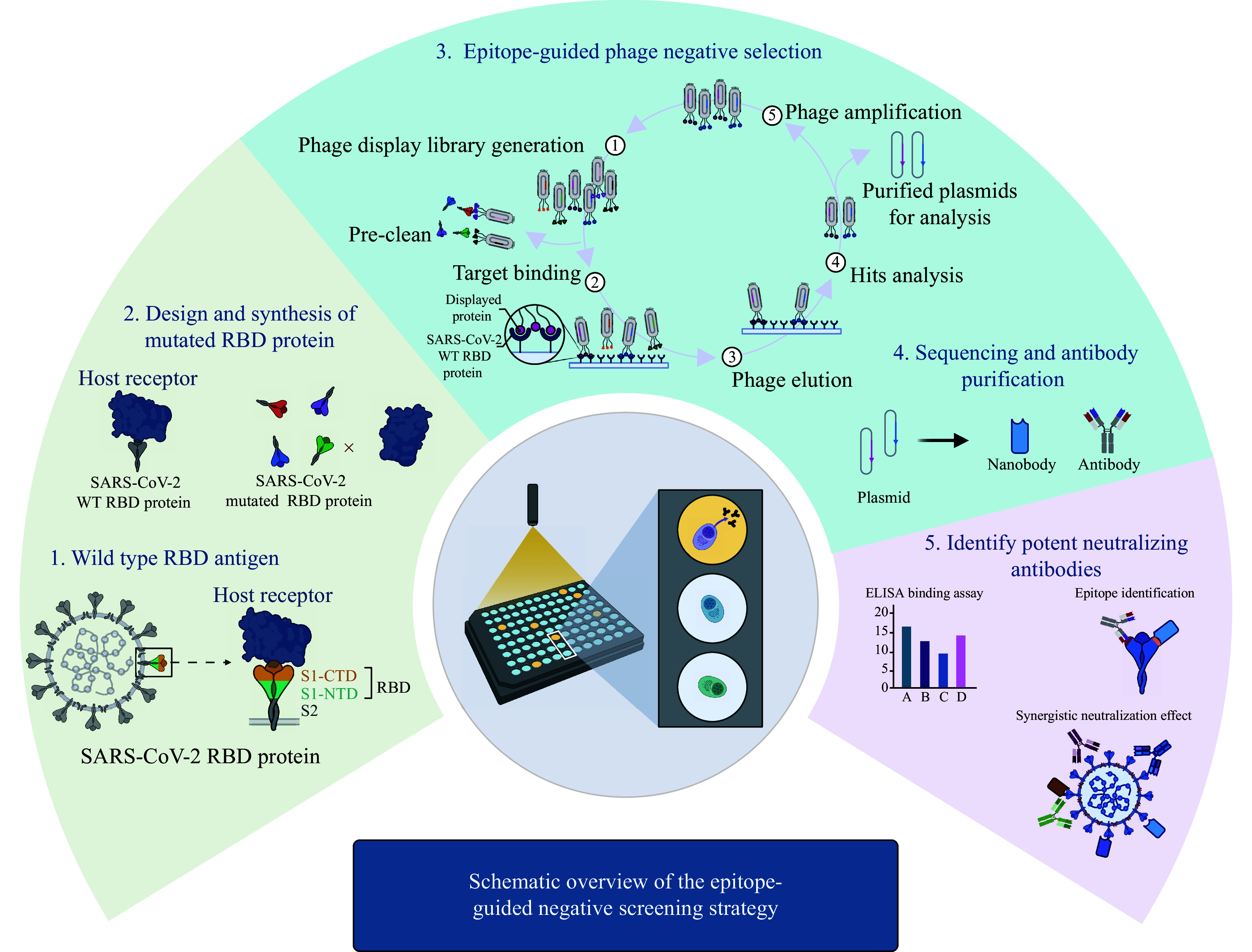
Schematic overview of the epitope-guided negative screening strategy. The workflow illustrates the key innovative step of using a mutated RBD antigen to perform negative screening against a phage display library. This process depletes phages that bind to the off-target epitope, thereby enriching for phages displaying antibodies that target the desired neutralizing epitope on the wild-type RBD. The enriched pool is then subjected to traditional positive selection and subsequent characterization of antibodies through binding (ELISA) and functional (neutralization) assays. Abbreviations: RBD, receptor-binding domain; SARS-CoV-2, severe acute respiratory syndrome coronavirus 2; WT, wild-type.

The effect of therapeutic antibodies depends on complex interactions between the antibodies, their targets, and associated effector molecules. Targeting specific epitopes of the antigen can significantly enhance the biological activity of antibodies and broaden their applications^[23]^. In this study, we established a standardized *in vitro* antibody production strategy by using epitope-guided phage display technology to efficiently screen therapeutic antibodies that bind to specific antigen epitopes (***Fig. 7***).

SARS-CoV-2 was selected as the target pathogen for antibody screening in the present study. The RBD of its spike protein directly binds to the cellular receptor ACE2 to initiate infection. We introduced mutations into the RBD and used the wild-type RBD as a positive antigen and the mutant RBD as a negative antigen. From various antibody libraries, we identified nine domain antibodies and one scFv antibody, and successfully purified multiple neutralizing antibodies targeting the RBD. *In vitro* experiments demonstrated that most of these antibodies exhibited strong antiviral activity by blocking ACE2 recognition. Since the SARS-CoV-2 outbreak, the virus has shown significant mutational diversity, with variants harboring mutations that enhance infectivity^[28]^. However, most of the antibodies we identified retained broad-spectrum neutralizing activity against variants such as G476S, V483A, and D614G. The SARS-CoV-2 spike protein is a trimer composed of S1 and S2 subunits^[29–30]^, and its transition from a closed to an open conformation within the trimer is essential for receptor binding and membrane fusion^[31–33]^. Therefore, antibodies that block the ACE2 binding interface in both states may be more effective in preventing infection. One of our antibodies, 4A12, appears to neutralize the virus through this mechanism, although further structural analysis is needed.

In antibody-based targeted therapies, using a single antibody often fails to achieve both safety and efficacy. As our understanding of the immune system and antibody engineering has advanced, therapies have progressed from classical monoclonal antibodies to more complex multi-antibody structures, known as "cocktail therapies"^[34]^. Using the epitope-guided phage display screening strategy developed in the present study, we rapidly identified several antiviral neutralizing antibodies. These antibodies target complementary epitope regions on the SARS-CoV-2-RBD. *In vitro* experiments demonstrated that antibodies recognizing different epitopes (4A12, 4A10, 4D5, 4D8 targeting epitope 1; 4A3 and 4C5 targeting epitope 2) exhibited synergistic antiviral effects, suggesting that our epitope-guided negative screening method is a safe, efficient, and rapid *in vitro* strategy for antibody engineering.

The correlation between antibody library size and successful selection is well established^[35–36]^. Although our naïve phage display library generated moderate-affinity antibodies (KD = 1–5.9 nmol/L), their neutralization potency remained lower than that of convalescent/camelid-derived equivalents due to limited complementarity-determining region (CDR) diversity. To address this, future work will implement structure-guided affinity maturation using AlphaFold2-predicted nanobody-RBD complexes to identify optimization hotspots (*e.g.*, Gly100/Gly103 in 4A12 CDR-H3), combined with artificial intelligence-accelerated mutagenesis *via* AbLIFT for *in silico* CDR scanning^[37]^. We will further employ generative design (IgVAE) to create non-natural high-affinity variants, integrating predictions with epitope-guided phage display under escalating stringency to preserve specificity^[38]^. This pipeline aims for a greater than 100-fold affinity improvement (KD < 0.1 nmol/L), enhancing clinical potential through reduced dosing and resilience against viral escape^[39–42]^. Notably, recent *in silico* studies have demonstrated that rational chimeric antibody design, which grafts optimized CDRs onto stable frameworks, effectively neutralizes immune-evasive variants like Omicron^[43–44]^, aligning with our computational engineering strategy.

In conclusion, we developed an epitope-guided phage display screening method that effectively and safely enables the rapid *in vitro* production of large quantities of specific therapeutic antibodies. Beyond identifying antiviral neutralizing antibodies, this strategy can be broadly applied to isolate functional blocking antibodies targeting various antigens, thereby inhibiting critical domains relevant to clinical development.

## SUPPLEMENTARY DATA

Supplementary data to this article can be found online.
