# Supplementary material for: Isolation of neutralizing antibodies against SARS-CoV-2 through an epitope-guided negative screening by phage display
Source: J Biomed Res. 2026 Mar 19;40(2):210–23. doi: 10.7555/JBR.39.20250259 (PMC13044406; doi:10.7555/JBR.39.20250259)
Supplement: Supplementary file 1 — Supplementary data to this article can be found online. [file jbr-40-2-210-S1.pdf]

Available online at www.jbr-pub.org.cn
Open Access at PubMed Central
Journal of Biomedical Research, 2026 40(2): 1–4
Supplementary Data
JBR

Isolation of neutralizing antibodies against SARS-CoV-2 through an epitope-guided negative screening by phage display

Ming Lu[1,△], Yin Chen[2,△], Xiaoyu Liu[1], Fang Gao[1], Liming Gou[1], Wei Ye[1], Jiaqi Wen[1], Xiling Guo[2,✉], Wei Gao[1,3,✉]

[1]*National Health Commission Key Laboratory of Antibody Technique, School of Basic Medical Sciences, Animal Core Facility of Nanjing Medical University, Nanjing Medical University, Nanjing, Jiangsu 211166, China;*
[2]*Key Laboratory of Enteric Pathogenic Microbiology; Ministry of Health Institute of Pathogenic Microbiology; Jiangsu Province Center for Disease Control and Prevention, Nanjing, Jiangsu 211166, China;*
[3]*The Second People's Hospital of Changzhou, the Third Affiliated Hospital of Nanjing Medical University, Changzhou, Jiangsu 213000, China.*

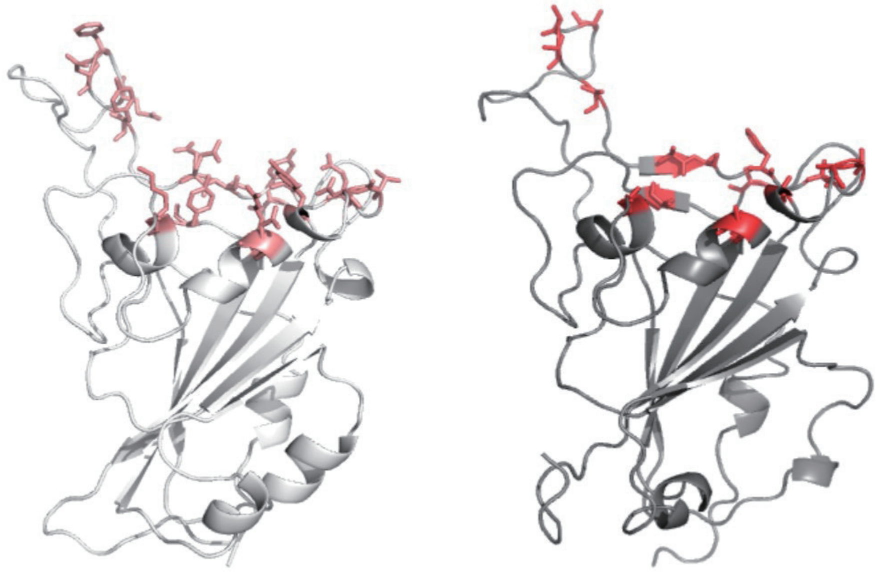

Supplementary Fig. 1 **Structural model of SARS-CoV-2-RBD mut.** The SARS-CoV-2-RBD mut structure model is established based on the SARS-CoV-2-RBD structure (PDB ID: 6M0J). The ACE2 interfaces of SARS-CoV-2-RBD and SARS-CoV-2 RBD mut are shown in light red stick and dark red stick, respectively. Abbreviations: RBD, receptor-binding domain; SARS-CoV-2, severe acute respiratory syndrome coronavirus 2.

△These authors contributed equally to this work.

✉Corresponding authors: Xiling Guo, Key Laboratory of Enteric Pathogenic Microbiology, Ministry of Health, Institute of Pathogenic Microbiology, Jiangsu Provincial Center for Disease Control and Prevention, Nanjing, Jiangsu 211166, China. E-mail: gxlnjjscdc@163.com; Wei Gao, National Health Commission Key Laboratory of Antibody Technique, School of Basic Medical Sciences, Animal Core Facility of Nanjing Medical University, Nanjing Medical University, 101 Longmian Avenue, Nanjing, Jiangsu 211166, China. E-mail: gao@njmu.edu.cn.

Received: 22 June 2025; Revised: 03 November 2025; Accepted: 06 November 2025; Available online: 10 November 2025; Published date: 19 March 2026

CLC number: R392.11, Document code: A

The authors reported no conflict of interests.

This is an open access article under the Creative Commons Attribution (CC BY 4.0) license, which permits others to distribute, remix, adapt and build upon this work, for commercial use, provided the original work is properly cited.

© 2026 by Journal of Biomedical Research.
https://doi.org/10.7555/JBR.39.20250259

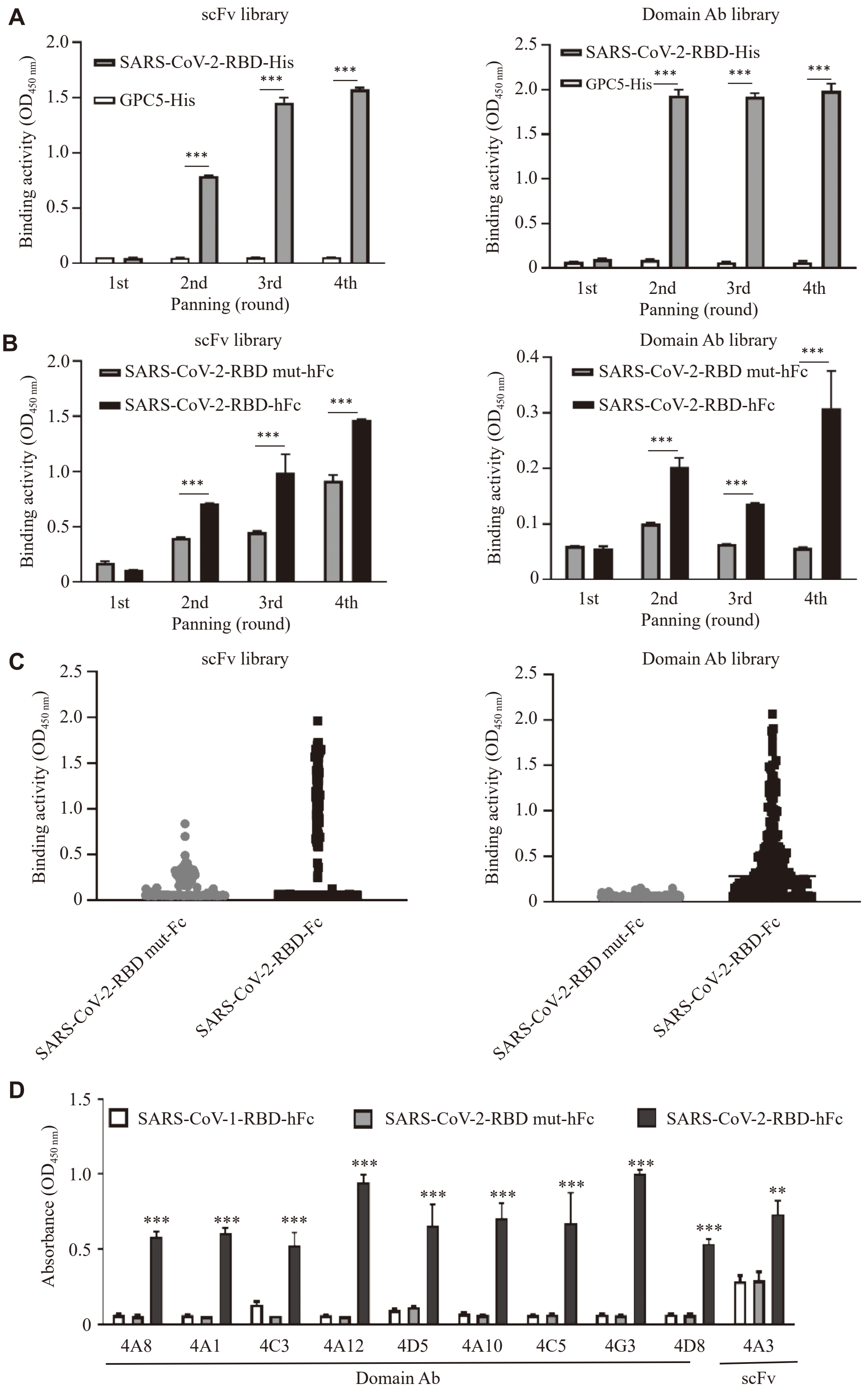

Supplementary Fig. 2 **Positive binder enrichment of antibody screening.** A: Polyclonal phage ELISA to detect the antigen-binding activity of four rounds of rescued phages. GPC5-his was used as a negative antigen control. B: Polyclonal phage ELISA to compare the binding activities of the eluted phage for SARS-CoV-2-RBD-hFc and SARS-CoV-2-RBD mut-hFc. C: Monoclonal phage ELISA to analyze SARS-CoV-2-RBD-specific binders. D: Capture phage ELISA to detect the antigen-binding specificity of soluble antibodies extracted from TG1 periplasm. Data are presented as the mean ± standard deviation (n = 3). Statistical significance was determined by one-way ANOVA with Tukey's multiple comparisons test. $^{**}P < 0.01$ and $^{***}P < 0.001$ indicate significant differences compared to the GPC5-His (A) or compared to the SARS-CoV-2-RBD mut-hFc group (B, D). Abbreviations: ELISA, enzyme-linked immunosorbent assay; RBD, receptor-binding domain; SARS-CoV-2, severe acute respiratory syndrome coronavirus 2.

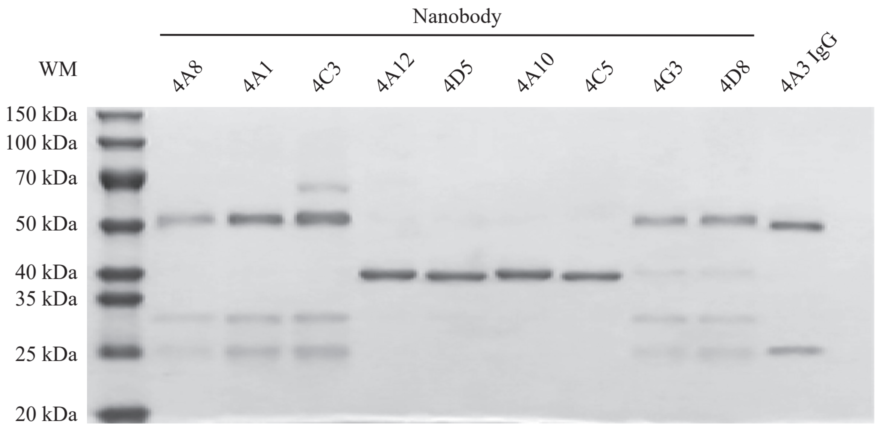

Supplementary Fig. 3 **SDS-PAGE of purified antibodies.** The purified IgG antibody (4A3) and nanobodies (4A8, 4A1, 4C3, 4A12, 4D5, 4A10, 4C5, 4G3, 4D8) were separated by SDS-PAGE and visualized by Coomassie Brilliant Blue staining. The positions of molecular weight markers are indicated on the left. Abbreviations: SARS-CoV-2, severe acute respiratory syndrome coronavirus 2; SDS-PAGE, sodium dodecyl sulfate-polyacrylamide gel electrophoresis.

Supplementary Table 1 **Antibody sequences and complementarity-determining region information**

Antibody	CDR-H1 (AA)	CDR-H2 (AA)	CDR-H3 (AA)	Sequences
4A3	GFTFSSYA	SIASSGYYTD	KDADS	EVQLLESGGGLVQPGGSLRLSCAASGFTFSSYAMSWVRQA PGKGLEWVSSIASSGYYTDYADSVKGRFTISRDNSKNTLY LQMNSLRAEDTAVYYCAKDADSFDYWGQGTLVTVSSGG GGSGGGGSGGGGSDIQMTQSPSSLSASVGDRVTITCRASQS ISSYLNWYQQKPGKAPKLLIYAASYLQSGVPSRFSGSGSGT DFTLTISSLQPEDFATYYCQQAYSAPSTFGQGTKVEIK
4A10	SFDFSDY	IGEIHHSGSTYYN PSLKSRV	VKDFVVGETAEFSY	EVQLLESGGGLVQPGGSLRLSCAASSFDFSDYEMSWVRQA PGKGLEWIGEIHHSGSTYYNPSLKSRVTISRDNSKNTLYLQ MNSLRAEDTATYYCVKDFVVGETAEFSYWGQGTLVTVSS
4A12	DFAFSSY	IGEIHHSGSTYYN PSLKSLV	VKDFGHLGQMAS	EVQLLESGGGLVQPGGSLRLSCAASDFAFSSYEMSWVRQA PGKGLEWIGEIHHSGSTYYNPSLKSLVTISRDNSKNTLYLQ MNSLRAEDTAVYYCVKDFGHLGQMASWGQGTLVTVSS
4C5	DFYFADY	IGSIYHSGSTYYN PSLKSRV	AREWHSGYDY	EVQLLESGGGLVQPGGSLRLSCAASDFYFADYEMSWVRQ APGKGLEWIGSIYHSGSTYYNPSLKSRVTISRDNSKNTLYL QMNSLRAEDTAMYYCAREWHSGYDYWGQGTLVTVSS
4D5	SFDFSSY	IGEIHHSGSTYYN PSLKSRV	VKDLGFADH	EVQLLESGGGLVQPGGSLRLSCAASSFDFSSYEMSWVRQA PGKALEWIGEIHHSGSTYYNPSLKSRVTISRDNSKNTLYLQ MNSLRAEDTAMYYCVKDLGFADHWGQGTLVTVSS
4D8	DFDFYDY	IGEIHHSGSTYYN PSLKSRV	VKDFVGADGPFVFDY	QVQLVQSGGGLVQPGGSLRLSCAASDFDFYDYEMSWVRQ APGKALEWIGEIHHSGSTYYNPSLKSRVTISRDNSKNTLYL QMNTLRAEDTAIYYCVKDFV GADGPFVFDYWGQGTLVTVSS

Supplementary Table 2 **Affinity measurement of SARS-CoV-2 receptor-binding domain to antibodies**

Ligand	Analyte	*ka* (1/Ms)	*kd* (1/s)	KD (nmol/L)	R_{max} (RU)	Chi² (RU²)
4A3 IgG	RBD protein	1.91E+06	6.90E−03	3.62	25.55	0.379
4A10	RBD protein	4.48E+05	4.61E−04	1.03	20.86	0.38
4A12	RBD protein	4.29E+05	1.06E−03	2.47	18.52	0.302
4D5	RBD protein	2.98E+05	1.74E−03	5.82	24.33	0.304

Abbreviation: SARS-CoV-2, severe acute respiratory syndrome coronavirus 2.

Supplementary Table 3 **Long-term SARS-CoV-2-neutralizing effects of candidate antibodies**

Antibodies	Concentration (μg/mL)	Exposure time (days)	Protection
4A3	10	10	100%
4A12	10	10	100%
4D5	37.5	15	100%

Abbreviation: SARS-CoV-2, severe acute respiratory syndrome coronavirus 2.

Supplementary Table 4 **Mutated residues of SARS-CoV-2-RBD mut**

SARS-CoV-1	SARS-CoV-2	SARS-CoV-2 mut
K390	R403	A403
D392	D405	A405
V404	K417	A417
Y436	Y449	A449
Y440	Y453	A453
P470	E484	A484
L472	F486	A486
N473	N487	A487
Y475	Y489	A489
N479	Q493	A493
D480	S494	A494
G482	G496	F496
Y484	Q498	A498
T486	T500	A500
T487	N501	A501
Y491	Y505	A505

Abbreviation: SARS-CoV-2, severe acute respiratory syndrome coronavirus 2.

Supplementary Table 5 **Mutations for mapping the epitope of SARS-CoV-2 nAbs**

Mutations for mapping the epitope of SARS-CoV-2 nAbs					
345T/346R	408R/409Q	446G/447G	467D/468I	481N/482G	496G/498Q
348A/349S	415T/416G	448N/449Y	469S/470T	483V/484E	499P
351Y/352A	417K/421Y	450N/452L	471E/472I	485G/486F	500T/501N
403R	437N	453Y	473Y/474Q	487N/489Y	502G/505Y
404G	439N/440N	455L/456F	475A/476G	490F	506Q
405D/406E	441L/442D	457R	477S/478T	493Q/494S	508Y
407V	444K/445V	458K/459S	479P	495Y	

Abbreviation: SARS-CoV-2, severe acute respiratory syndrome coronavirus 2.
